# Complete Genome Sequence of an ON1 Human Respiratory Syncytial Virus Strain Isolated in Lebanon in 2015

**DOI:** 10.1128/genomeA.00316-18

**Published:** 2018-04-19

**Authors:** Amani Ezzeddine, Nadia Soudani, Chun Kiat Lee, Ghassan Dbaibo, Husni Elbahesh, Hassan Zaraket

**Affiliations:** a Department of Experimental Pathology, Immunology and Microbiology, Faculty of Medicine, American University of Beirut, Beirut, Lebanon; b Center for Infectious Diseases Research, Faculty of Medicine, American University of Beirut, Beirut, Lebanon; c Department of Laboratory Medicine, National University Health System, Singapore; d Department of Pediatrics and Adolescent Medicine, Faculty of Medicine, American University of Beirut Medical Center, Beirut, Lebanon; e Department of Biochemistry and Molecular Genetics, Faculty of Medicine, American University of Beirut, Beirut, Lebanon; f Department of Microbiology, Immunology and Biochemistry, University of Tennessee Health Science Center, Memphis, Tennessee, USA

## Abstract

We report here the complete genome sequence of a human respiratory syncytial virus (HRSV) strain obtained from an infant who presented to the emergency room with an acute respiratory illness during the 2014/2015 HRSV season in Lebanon. Analysis revealed that this virus belongs to the ON1 genotype that has recently emerged worldwide.

## GENOME ANNOUNCEMENT

Human respiratory syncytial virus (HRSV) is a major cause of acute lower respiratory infections (ALRIs) in children worldwide. The clinical outcomes vary from mild respiratory tract infections to severe bronchiolitis and pneumonia (1). Annually, more than 30 million episodes of HRSV-associated ALRIs occur globally in children under 5 years of age (1). Currently, there is no available vaccine against HRSV. Genome-based characterization of HRSV allows for better understanding of its global diversity and provides information for the development of vaccines, antivirals, and diagnostics (2). HRSV is a member of the family Paraxymoviridae and subfamily Pneumovirinae (3). It is an enveloped virus with a 15-kb negative-sense single-stranded RNA genome that encodes 11 viral proteins (3). HRSVs are divided into two groups (A and B) based on their antigenic variability. Thirteen HRSV-A genotypes and 20 HRSV-B genotypes have been identified on the basis of the G protein diversity (4). HRSV-A infections are more common than HRSV-B infections. This antigenic variability contributes to the repeated infections that occur throughout life (5).

The isolate RSVA/Lebanon/14LJF289/2015 (JF289) was collected in Lebanon from an infant who presented to the emergency room with an acute respiratory illness during the 2014/2015 HRSV season. The patient had no history of palivizumab use. RNA extraction was performed using a Purelink virus RNA/DNA kit followed by first-strand cDNA synthesis with an RSV-specific primer and SuperScript III reverse transcriptase. Purification of the cDNA was performed using Agencourt AMPure XP beads followed by double-stranded DNA synthesis with a Sigma SeqPlex XE kit. The libraries were prepared with a NEBNext fast library prep kit, and ion sphere particles were generated using the Ion OneTouch 2 system. Sequencing was carried out on the Ion Proton sequencer. The obtained sequences were trimmed and assembled using Geneious software, and the gaps were filled by using the primer-walking method and Sanger sequencing.

The genome of JF289 contains 15,098 nucleotides, including a 3′ leader and 5′ trailer. The 11 open reading frames (ORFs) encoding viral proteins include two nonstructural proteins (NS1and NS2), a nucleocapsid protein (N), a matrix protein (M), a phosphoprotein (P), an attachment protein (G), a fusion protein (F), a small hydrophobic protein (SH), transcription elongation factors (M2-1 and M2-2), and a large protein (L). The contents of A, U, G, and C were 38.91%, 27.69%, 15.65%, and 17.74%, respectively. BLAST analysis revealed the highest similarity with RSVA/USA/TH_10323/2013 with 99% identity. JF289 possessed an N276S substitution in the F protein, which has been associated with partial resistance to palivizumab and may lead to further mutations leading to complete resistance (6, 7).

The sequence of the G protein was aligned with other HRSV-A sequences retrieved from GenBank, and a phylogenetic tree was constructed using the neighbor-joining method with MEGA version 6.0. Based on the tree analysis, JF289 belongs to the recently identified ON1 genotype that has emerged globally since 2010. ON1 has a 72-nucleotide duplication within the G protein’s second hypervariable region. The increase in frequency of the ON1 genotype worldwide suggests an ability of this genotype to escape host immunity and sustain transmission.

### Accession number(s).

The complete genome of the RSVA/Lebanon/14LJF289/2015 virus was deposited in GenBank under the accession number MG793382.
